# All Eggs Are Not Equal: The Maternal Environment Affects Progeny Reproduction and Developmental Fate in *Caenorhabditis elegans*

**DOI:** 10.1371/journal.pone.0025840

**Published:** 2011-10-04

**Authors:** Simon C. Harvey, Helen E. Orbidans

**Affiliations:** Ecology Research Group, Department of Geographical and Life Sciences, Canterbury Christ Church University, Canterbury, United Kingdom; Centre National de la Recherche Scientique & University of Nice Sophia-Antipolis, France

## Abstract

**Background:**

Maternal effects on progeny traits are common and these can profoundly alter progeny life history. Maternal effects can be adaptive, representing attempts to appropriately match offspring phenotype to the expected environment and are often mediated via trade-offs between progeny number and quality. Here we have investigated the effect of maternal food availability on progeny life history in the free-living nematode *Caenorhabditis elegans*.

**Methodology/Principal Findings:**

The maternal environment affects both reproductive traits and progeny development. Comparisons of the progeny of worms from high and low maternal food environments indicates that low maternal food availability reduces progeny reproduction in good environments, increases progeny reproduction in poor environments and decreases the likelihood that progeny will develop as dauer larvae. These analyses also indicate that the effects on progeny are not a simple consequence of changes in maternal body size, but are associated with an increase in the size of eggs produced by worms at low maternal food availabilities.

**Conclusions/Significance:**

These results indicate that the maternal environment affects both progeny reproduction and development in *C. elegans* and therefore that all progeny are not equal. The observed effects are consistent with changes to egg provisioning, which are beneficial in harsh environments, and of changes to progeny development, which are beneficial in harsh environments and detrimental in benign environments. These changes in progeny life history suggest that mothers in poor quality environments may be producing larger eggs that are better suited to poor conditions.

## Introduction

Maternal effects are widespread in natural and experimental populations [1], [2] and can have pervasive effects on population dynamics [3]. Many maternal effects are a response to unfavorable conditions and are often mediated via trade-offs between progeny number and aspects of progeny quality [4]. These trade-offs can represent attempts to appropriately match progeny phenotype to the expected progeny environment [1], [4], [5]. For example, in *Daphnia*, poor maternal conditions often result in the production of a smaller number of larger eggs, with progeny subsequently performing better under a range of stressful conditions (*e.g.* [6]–[10]). Other effects can be more complex, producing changes to offspring life history that are not directly related to resource provisioning (*e.g.* [11]) or that interact in complex ways with the direct effects of conditions on the mother [12]–[14].

Here, the effects of the maternal environment on progeny reproduction and development are investigated in the free-living nematode *Caenorhabditis elegans*. In the wild, *C. elegans* is associated with nutrient- and bacteria-rich substrates [15]–[17]. Dispersal between these ephemeral resource patches is undertaken by an alternative third larval stage, the developmentally arrested and long lived dauer larvae [18]–[20]. Growing populations can reach very large sizes, and it is likely that *C. elegans* routinely experiences very high population densities and has therefore evolved to optimize fitness, *i.e.* population growth and/or the production of dispersal stages, under such conditions.

Extensive studies of individual worms have defined development and reproduction in *C. elegans* (*e.g.* [21]–[25]). However, population sizes predicted from individual worm data significantly overestimate observed population sizes, indicating that there are strong density dependent effects on population growth in *C. elegans* [24]. The observed food concentration-dependent reduction in fecundity seen in *C. elegans* partly underlies this discrepancy [24], but does not fully explain it. One possible explanation is that, in addition to the direct effects on worms that are seen under poor conditions, there are also changes in the progeny produced by worms in poor conditions. Understanding how *C. elegans* responds to poor conditions is important in understanding the ecology and evolutionary biology of the species, and will also allow the molecular and genetic bases of such effects to be investigated in this important model species.

Here we show, for the first time, that maternal resource limitation affects progeny reproduction and development in *C. elegans*. These effects are shown to be not directly related to changes in maternal body size, but are associated with changes in egg size. These changes are consistent with an adaptive explanation, whereby low maternal food availabilities result in fewer larger eggs and progeny that are better adapted to poor conditions.

## Results

### Maternal food availability affects progeny reproductive traits

**Figure 1:**
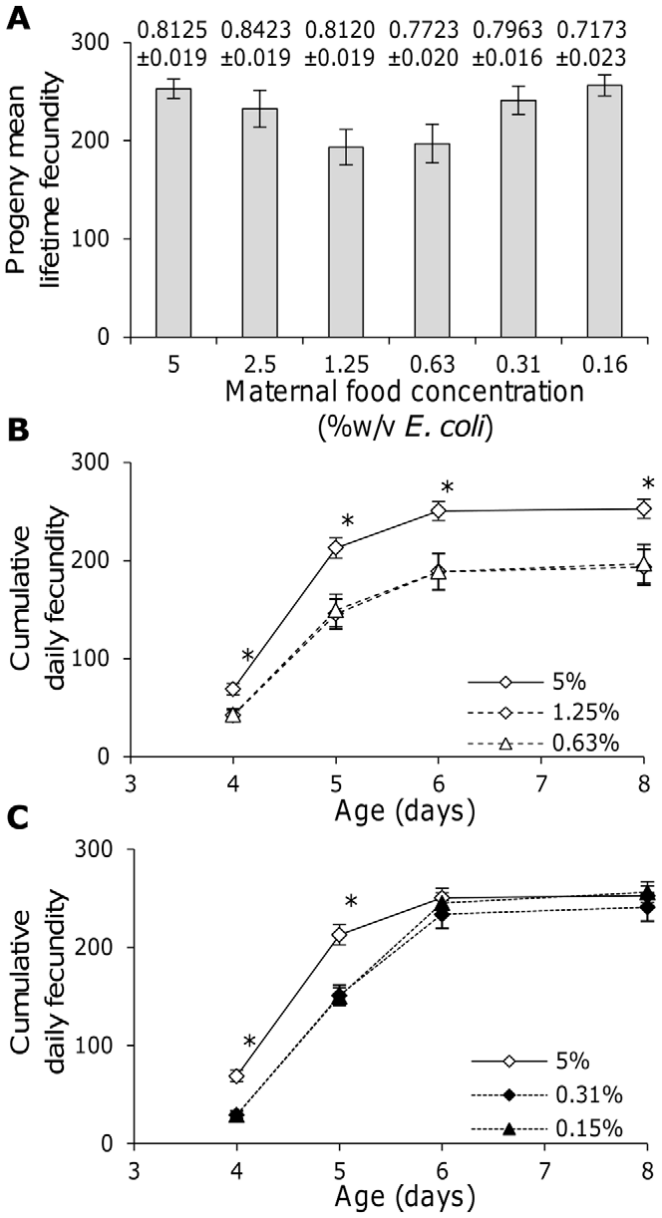
Maternal food availability affects progeny reproduction. Lifetime fecundity (A) and mean cumulative daily fecundity (B and C) on *ad libitum* food of the progeny of worms from different maternal food availabilities. Error bars are ±1 standard error and the mean (±1 standard error) maternal length for each food availability is noted above the corresponding bar in A. * Indicates that the cumulative progeny fecundity on that day differs significantly between parental food availabilities (Kruskal-Wallis, *p*<0.05). Mean fecundities were calculated from at least 21 hermaphrodites (range 21–28, median of 26).

To investigate the effects of maternal food availability on progeny reproductive traits, eggs were isolated from early adult worms grown at differing food availabilities and progeny were allowed to develop at *ad libitum* food conditions. In these conditions, progeny lifetime fecundity (Figure 1A: F_5, 148_ = 2.69, *p* = 0.017) and reproductive schedule was affected by maternal food availability (Figure 1B & C), with progeny from high and very low maternal food availabilities having higher lifetime fecundity than progeny from mothers at intermediate food levels. Progeny from the highest maternal food concentrations also showed the greatest early reproduction (Figure 1 B & C), while progeny from very low maternal food concentrations showed greater late fecundity (Figure 1C). As only limited numbers of eggs could be isolated from mothers at the lowest food concentrations used here, these changes could not be analyzed in greater detail. However, the changes in the reproductive schedule of the progeny from very low maternal food concentrations would slow their expected rate of population growth. These data indicate that maternal conditions can affect progeny reproductive traits in *C. elegans*.

### Maternal size does not fully explain variation in progeny reproductive traits

Fecundity is often found to increase with body size and in the nematoda there is generally a close linkage between adult female body size and fecundity [26]. Therefore, the influence of maternal body size on progeny reproduction was investigated. Progeny were isolated from individual adult worms from three maternal food treatments at each of two maternal age groups. Here, maternal food treatments were selected at the point of progeny isolation from a wider range of food concentrations. This was done to account for variation between experiments in batches of *E. coli* and in the maternal *per capita* food availability. High maternal food availability represented *ad libitum* food and excess food remained on these plates. Low maternal food availability represented the lowest food concentration from which sufficient progeny could be isolated and no food remained on these plates. An intermediate food concentration, where small quantities of food remained on the plates, was then selected to represent Medium maternal food availability. Analysis of maternal size indicated that, as expected, older mothers were larger (Age: F_1, 43_ = 144.6, *p*<0.001) and that decreased food availability reduced maternal size (Food: F_2, 43_ = 64.7, *p*<0.001). There was no interaction between maternal food availability and maternal age (Food×Age: F_2, 43_ = 0.7, *p* = 0.53). This indicates that the maternal size did differ across the High, Medium and Low food concentrations chosen and that it did so in a similar way in the two maternal age groups.

**Figure 2:**
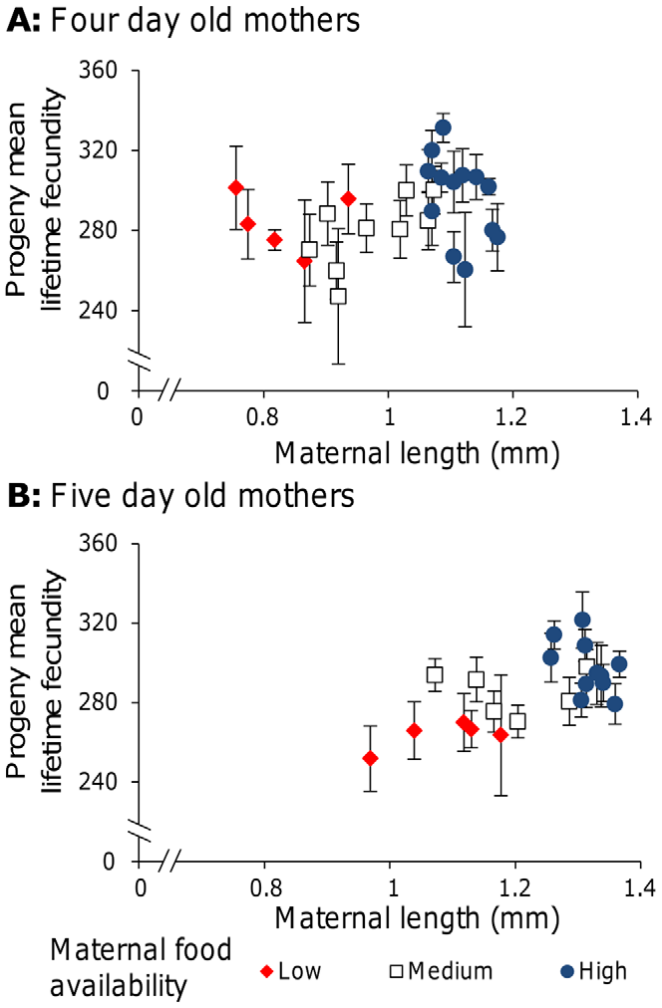
Maternal size does not explain variation in progeny reproduction. The mean lifetime fecundity of the progeny of individual four (A) and five (B) day old parents that developed at High food (20% w/v in both cases), where excess food remained, Low food (1.25% and 2.5% w/v for four and five day old worms, respectively), where food had been depleted, and Medium food (2.5% and 5% w/v for four and five day old worms, respectively), where small quantities of food remained. Note that food concentrations differed between age groups due to the differing times that worms had been maintained on the plates. Mean fecundities were calculated from 4–10 progeny/parent (median 8).

There was a positive relationship between maternal size and mean progeny lifetime fecundity for five day old mothers (Figure 2B, Pearson product-moment correlation: *r* = 0.63, *p* = 0.002), but not for four day old mothers (Figure 2A, Pearson product-moment correlation: *r* = 0.32, *p* = 0.10). Analysis of early reproduction indicated that there was a positive relationship between maternal size and mean progeny fecundity on the first day of reproduction for both the 4 and 5 day old worms (Pearson product-moment correlation: *r* = 0.45, *p* = 0.03 and *r* = 0.44, *p* = 0.02, respectively). These data indicate that there is a link between maternal size and offspring reproduction, with larger mothers producing progeny that start reproduction earlier and tend to have higher lifetime fecundity. However, with maternal size fitted as a covariate, analysis of progeny lifetime fecundity indicated that, while maternal age did not alter progeny fecundity (Age: F_1, 363_ = 0.08, *p* = 0.80), decreased maternal food availability did reduce progeny lifetime fecundity (Food[Age]: F_4, 363_ = 3.32, *p* = 0.01). Variation in maternal size does not therefore fully explain the differences in progeny reproduction.

The potential role of maternal size in determining progeny reproductive traits was also investigated in worms grown at different temperatures and in worms exposed to a mild heat stress (Table 1). These data indicate that while maternal growth temperature does affect progeny lifetime fecundity, it is the progeny of the smaller parents that have higher lifetime fecundity. In the analysis of the effects of heat stress, maternal body size was affected, while progeny reproductive traits are not. These data support the view that maternal body size is not causing the observed variation in progeny reproductive traits.

**Table 1:** Maternal size and progeny reproduction in worms grown at differing temperatures and exposed to heat stress.

			Progeny	
	Treatment	Maternal length ± se (mm)	Lifetime fecundity ± se	Reproductive schedule
Temperature	15°C	0.940±0.005^a^	280.36±9.02^a^	NS*
	20°C	0.954±0.004^b^	248.64±8.15^b^	
Thermal stress	Control	1.226±0.011^a^	261.04±9.82	NS
	Heat shock	1.170±0.013^b^	262.72±9.00	

Superscript letters indicate groups that differ significantly in either maternal length or progeny lifetime fecundity (*p*<0.05), NS indicates no significant difference between treatments.

*The daily fecundity was higher for the progeny of the parents grown at 15°C worms on each day, but these differences were not significant.

### Maternal food availability affects progeny development

**Figure 3:**
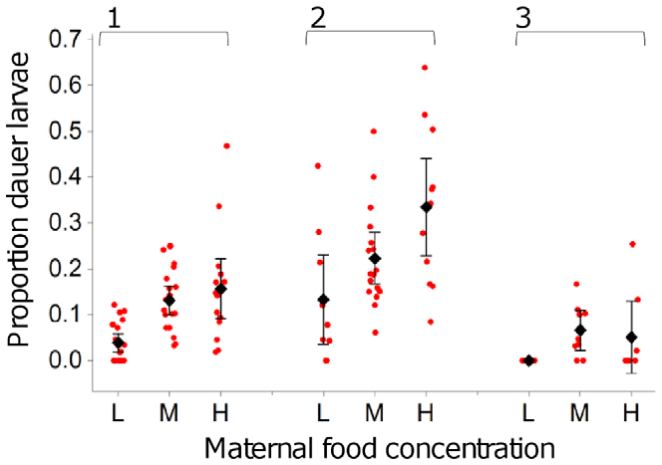
Maternal food availability affects development fate. Jitter plot showing the proportions of the L1 progeny of mothers from high (H), medium (M) and low (L) maternal food concentrations that developed as dauer larvae in three independent experiments (1–3). The mean proportion (±95% confidence interval) of L1 progeny that developed as dauer larvae is also shown for each treatment. In all cases High maternal food availability was 10% w/v *E. coli*. Medium maternal food availability was 2.5% w/v *E. coli* for assays 1 and 3, and a combination of 2.5% and 5% w/v *E. coli* treatments for assay 2. Low maternal food availability was 1.25% w/v *E. coli* for assays 2 and 3, and a combination of 1.25% and 0.625% w/v *E. coli* treatments for assay 2. Mean proportions were calculated from 8–20 plates/treatment (median 12).

Developing *C. elegans* larvae exposed to conditions unfavorable for growth and reproduction will arrest development as dauer larvae [18]–[20]. To investigate if maternal food availability affects the likelihood that progeny develop as dauer larvae, the arrested L1 progeny from differing maternal food availabilities were assayed for dauer larvae development (Figure 3). Analysis of these data indicated that dauer larvae development is affected by maternal food availability (H = 21.31, d.f. = 2, *p*<0.001, H = 9.99, d.f. = 2, *p* = 0.007 and H = 7.88, d.f. =  , *p* = 0.019 for assays 1, 2 and 3, respectively), with decreased maternal food availability reducing the proportion of progeny that developed into dauer larvae (Figure 3).

A lower likelihood of dauer larvae development in the progeny of worms from a poor environment could imply that these worms are better suited to growth and reproduction in poor environments. This would imply that changes in progeny life history are adaptive, representing an attempt to increase progeny fitness in poor environments. Alternatively, if progeny are low quality and dauer larvae development is affected by body condition, a common observation in dispersal decisions [27], then this could explain the reduced likelihood of dauer larvae formation. To distinguish between these possibilities, the effects of maternal food availability on progeny reproductive traits were investigated when progeny were allowed to develop in poor conditions.

### Changes in progeny life history are consistent with an adaptive explanation

**Figure 4:**
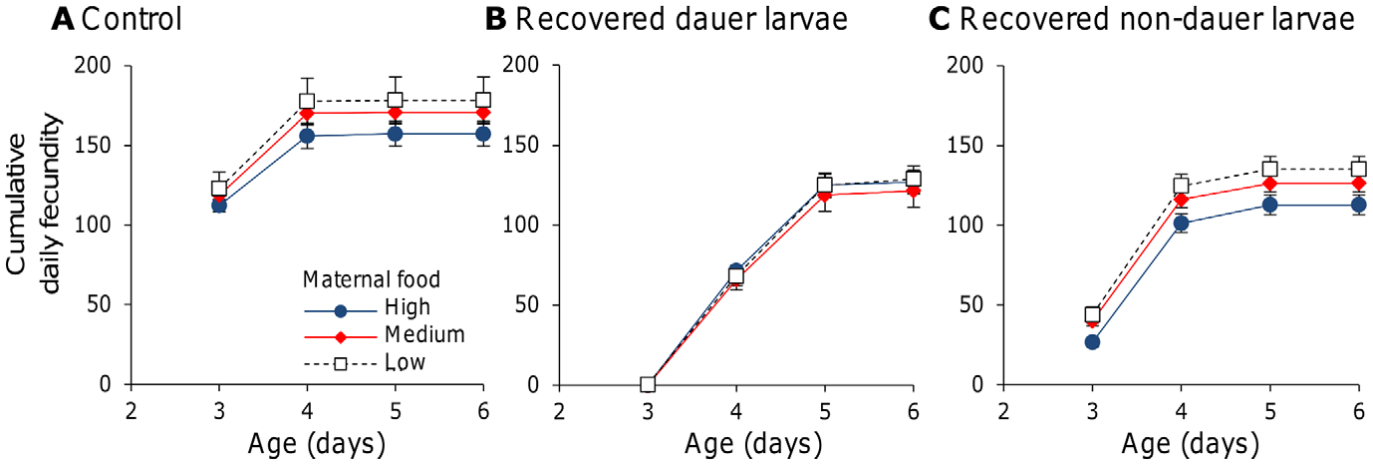
Effects of maternal condition depend on progeny environment. The mean cumulative daily fecundity at 25°C of (A) control, *ad libitum* food, progeny, (B) recovered dauer larvae progeny and (C) recovered non-dauer larvae progeny of mothers from a range of maternal food concentrations. Recovered dauer and non-dauer larvae were isolated from a standard dauer larvae assay prior to the onset of reproduction. High maternal food availability was 10% w/v *E. coli*, Medium maternal food availability was 2.5% w/v *E. coli* and Low maternal food availability was 1.25% w/v *E. coli*. Fecundities were calculated from at least 16 hermaphrodites (range 16–40, mean of 24.2). Error bars are ±1 standard error.

To investigate the effects of maternal food availability on progeny reproductive traits when progeny developed in a poor quality environment, worms were recovered from a dauer development assay and their reproductive traits then investigated. Three different maternal food availabilities were investigated and three progeny treatments were analyzed: dauer and non-dauer larvae recovered from a dauer larvae development assay and control worms that had developed at *ad libitum* food. As dauer development assays are performed at 25°C, fecundity was also analyzed at this temperature and this experiment therefore included two separate stresses. Firstly, all progeny treatments were grown and assayed at 25°C, a temperature at upper limit of the thermal niche that greatly reduces lifetime fecundity [21], [23]. Secondly, the dauer and non-dauer larvae recovered from the dauer development assay developed with limited food and high dauer pheromone levels, conditions that are similar to what would be expected in late stage growing populations of *C. elegans*. This therefore represents the conditions that the progeny of food restricted mothers might be expected to experience in the wild. Lifetime fecundity was reduced in worms recovered from the dauer assays in comparison to that in the control worms (Figure 4), indicating that the conditions in the dauer development assays imposed a further stress above that of development at 25°C. Maternal food availability did not affect lifetime fecundity in control worms, recovered dauer larvae or in recovered non-dauer larvae (Figure 4: F_2, 70_ = 1.05, *p* = 0.36, F_2, 56_ = 0.20, *p* = 0.83, F_2, 75_ = 2.32, *p* = 0.11, respectively). Similarly, there was no effect of maternal food availability on early reproduction in the control worms and recovered dauer larvae (Figure 4A & B: F_2, 70_ = 0.66, *p* = 0.52 and F_2, 56_ = 0.24, *p* = 0.79, respectively). This indicates that the reproductive advantages seen in the progeny of high food mother at 20°C is not seen when progeny are grown at 25°C. However, maternal food availabilities did affect early reproduction in recovered non-dauer larvae (F_2, 75_ = 5.02, *p* = 0.009), with progeny from high maternal food concentrations showing lower early reproduction (Figure 4C). The comparison of Figure 4A with Figures 4B and C indicates that the additional stress of having gone through a dauer development assay does affect the non-dauer worms (reduced early fecundity, Fig. 4C), but not the recovered dauer larvae (Fig. 4B). Therefore there is an effect of the conditions experienced during development and this is the reverse of the pattern observed in Figures 1 and 2. This therefore indicates that, under non-optimal, stressful conditions, the progeny of low food mothers do not perform worse than the progeny of high food mothers (Figure 4A & B) and that under certain conditions they perform better (Figure 4C).

**Figure 5:**
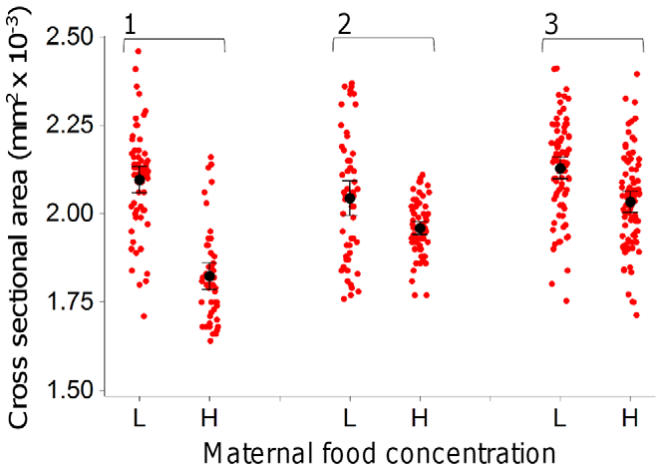
Maternal food availability affects egg size. Jitter plot showing the cross sectional area of eggs isolated from mothers grown at high (H) and low (L) maternal food concentrations in three independent experiments (1–3). The mean cross sectional area (±95% confidence interval) is also shown for each treatment. In all cases High maternal food availability was 10% w/v *E. coli*. Low maternal food availability was a combination of 2.5% and 0.125% w/v *E. coli* for assay 1, 0.3125% w/v *E. coli* for assay 2 and 1.25% w/v *E. coli* treatments for assay 3. Mean cross sectional areas were calculated from 49–83 eggs/treatment (median 64).

These data show that the effects of maternal food availability on progeny reproduction are reversed under poor conditions (Figures 1 & 2 vs. Figure 4). This supports the idea that the observed changes in progeny life history are adaptive. To further investigate this, the effect of maternal food availability on egg size was investigated. These assays indicated that maternal food availability did affect egg size (Figure 5, W = 4693.5, *p*<0.001, W = 3722.5, *p* = 0.016 and W = 7746.5, *p*<0.001 for assays 1, 2 and 3, respectively), with eggs from low maternal food availabilities being larger than those from high maternal food availabilities. These data indicate that egg size differs across maternal food environments, with low food mothers producing eggs that are, on average, larger than those of high-food mothers. However, variation in egg size within maternal food treatments is high, and eggs from low food mothers are also more variable in size than those from high food mothers (Figure 5).

## Discussion

Here we show that maternal environment changes progeny reproduction and development in *C. elegans* (Figures 1, 2, 3, 4 and Table 1). Many maternal effect genes, primarily identified by their ability to rescue embryonic lethal phenotypes, are known in *C. elegans* [28], and some maternal effect mutations alter progeny reproduction and developmental timing (*e.g.* [29]). This however represents the first demonstration of maternal effects on reproduction and development in non-mutant *C. elegans*, a finding with important implications for understanding and estimating fitness in *C. elegans*.

These data indicate that low maternal food availability can reduce progeny lifetime fecundity (Figure 1A & Figure 2) and delay progeny reproduction (Figure 1B & C) when progeny are allowed to develop in good environments. In contrast, when progeny are allowed to develop in poor environments, it is high maternal food availability that decreases early reproduction in progeny (Figure 4). Further, low maternal food availability is shown to decrease the likelihood that progeny will develop as dauer larvae (Figure 3). These observed effects of maternal conditions on progeny life history in *C. elegans* could represent a switch to the production of fewer, better provisioned eggs, in poor conditions and would mirror observations in *Daphnia* [6]–[10]. As such they would represent attempts to match progeny development to the expected environmental conditions and are therefore likely to be adaptive [1], [4].

Mechanistically it is not clear how the observed changes in progeny life history are produced. While maternal size is related to progeny reproduction, progeny lifetime fecundity is affected by the maternal environment when maternal size is controlled (Figure 2) and maternal body size and progeny reproductive traits can vary independently (Table 1). It is therefore clear that there is not a simple relationship between progeny quality and parental size. The observed increases in egg size at low maternal food availabilities (Figure 5) suggest that, assuming a direct link between egg size and quality, there could be differences in resource provisioning. However, maternal food availability also changes the number of eggs *in utero* and the developmental stage at which eggs are laid, which in turn will slightly change the duration of L1 arrest experienced by larvae from different maternal food treatments. It is therefore possible that these differences may affect progeny reproduction and development. Further, as progeny from low maternal food conditions do not perform best under all conditions (Figures 1 & 2 vs. Figure 4), it is unlikely that there is a simple relationship between egg size and progeny quality. Indeed, the data suggests that the observed effects may be a consequence of changes to egg provisioning, which are beneficial in harsh environments, and of changes to progeny development, which are beneficial in harsh environments and detrimental in benign environments. It would also be interesting to know how differences in progeny lifetime fecundity are produced. Given that lifetime fecundity in *C. elegans* under *ad libitum* food conditions is normally constrained by the number of sperm produced during the L4 stage [30], this suggests that either the duration or rate of spermatogenesis has been altered. Any such changes might then alter the onset of reproduction.

Given that very small changes (2–3 hours) in the onset of reproduction have dramatic effects on population growth rate in *C. elegans* [22] the observed changes in progeny life history will affect the properties of growing populations. Indeed, changes in early reproduction are likely to be particularly important for *C. elegans* in the wild given the greatly reduced lifespan observed under more natural conditions [31]. However, given the complex nature of the changes in progeny traits, and the changes in the likelihood that worms will develop as dauer larvae (Figure 3), it is difficult to predict how the dynamics of growing populations of *C. elegans* will be affected by the maternal effects observed here. Given this, it is a priority to determine how eggs differ in their composition, if differences in gene expression in developing larvae can be identified and to determine the mechanistic bases of changes in progeny reproductive traits.

## Materials and Methods

### Worms

Experiments were performed using the N2 isolate of *C. elegans*, obtained from the *Caenorhabditis* Genetics Center, which is funded by the NIH National Center for Research Resources (NCRR). Synchronized populations of L1s were produced by allowing eggs isolated from hypochlorite treated adults [32] to hatch on plates without food and to develop at 20°C for 24 hours. This causes worms to arrest development prior to the L1/L2 molt, a state in which they can survive for several weeks, and hence produces a cohort of worms at the same developmental stage. All treatments were randomized and blind coded, with plates on which worms failed to grow, burrowed into the agar or climbed the sides of the plates excluded from analyses.

### Maternal food availability and progeny reproduction

Arrested L1s were transferred, at 20°C, to plates containing a food source consisting of 100 µL of various w/v concentrations of OP50 *Escherichia coli* diluted in water. For these assays worms were grown on dauer agar plates [33], on which the *E. coli* food source cannot grow. After transfer to food, worms were allowed to develop for three days, by which time egg-laying had started, worms were then hypochlorite treated to isolate eggs [32]. The lifetime fecundity and reproductive timing of the arrested L1s that developed from these eggs was then determined by transferring worms individually to NGM agar plates with an excess of OP50 and allowing them to develop to adulthood. Once reproduction had started, worms were transferred to fresh plates daily until reproduction had ceased, with the number of larvae that developed on the plates from which the progeny had been removed counted to determine daily and lifetime fecundity [22]. Note that progeny spent the time from fertilization to egg isolation, estimated as a maximum of 2–3 hours, in the reproductive tract of mothers in differing environments. All data were analyzed using Minitab® Statistical Software (Mintab Ltd., Coventry). Lifetime fecundities were Johnson transformed and analyzed by ANOVA, with parental food concentration fitted as a factor. Progeny reproductive schedules were analyzed by comparing the daily fecundities using Kruskal-Wallis tests. Note that direct comparisons of specific % w/v food concentrations between experiments is not possible as the *per capita* food availability varied between experiments (*i.e.* the number of mothers per plate varied) and there are differences between batches of *E. coli* that affect worm life history.

### Maternal size and progeny reproduction

Worms from a single cohort of arrested L1s (grandmothers) were fed on two consecutive days and each population allowed to develop for three days on NGM plates with excess food. Eggs were then isolated and progeny allowed to arrest as L1s. These L1s (mothers) were then transferred to dauer agar plates [33] containing 100 µL food patches of various % w/v concentrations of OP50 *E. coli* diluted in water and allowed to develop until they were four or five days old (the staggered feeding of the grandmothers allowed both four and five day old mothers to be analyzed on the same day). To account for variation between batches of *E. coli* and for variation in the *per capita* food availability, maternal food treatments were selected at the point of progeny isolation from a wider range of food concentrations. The highest food concentration used in the assay was defined as High maternal food availability, and excess food remained on the plates in this treatment. Low maternal food availability was selected as the lowest food concentration from which sufficient numbers of progeny could be isolated and on these plates the bacterial food had always been depleted. An intermediate concentration, where small quantities of food remained on the plates, was then selected to represent Medium maternal food availability. Assessment and classification of maternal food treatments therefore occurred prior to progeny isolation and analysis. Once food treatments had been selected, mothers were individually transferred to fresh dauer agar plates without food, photographed using a Moticam 2000 video camera (Motic, Wetzlar, Germany) and parental body size, the length from the mouth to the base of the tail, determined in ImageJ [34]. Mothers were then transferred to a drop of hypochlorite solution to isolate fertilized *in utero* eggs (progeny), which were allowed to hatch and arrest as L1s. Progeny therefore differed in maternal age (4 day old vs. 5 day old mothers), maternal food availability (defined as High, Medium and Low, as described above) and in the length of time their grandmothers had spent in L1 arrest (1 vs. 2 days). As above, progeny also spent the time from fertilization to egg isolation in different environments. Lifetime fecundity and reproductive timing were then determined as described above. Here, High maternal food availability was 20% w/v *E. coli* for both the four and five day old mothers, Low maternal food availability was 1.25% and 2.5% w/v *E. coli* for four and five day old worms, respectively, and Medium maternal food availability was 2.5% and 5% w/v *E. coli* for four and five day old worms, respectively. Note that the differences between food concentrations for the four and five day old worms are a consequence of the additional day that the five day old mothers spent on the plates.

Maternal body size was analyzed by ANOVA, with Age and Food and the Age by Food interaction tested. Progeny lifetime fecundities were Johnson transformed and analyzed by ANOVA, with maternal Body Size fitted as a covariate, and maternal Age and maternal Food fitted as factors, with maternal Food nested within maternal Age. The relationship between maternal body size and progeny reproduction was investigated by correlating parental size with the mean number of progeny produced on the first day of reproduction and the mean progeny lifetime fecundity.

### Maternal growth temperature and progeny reproduction

Arrested L1s were fed on two consecutive days, with worms fed on day 1 maintained at 15°C and those fed on day 2 maintained at 20°C, and allowed to develop on NGM plates with excess food. Progeny were isolated and analyzed, as previously described, from all worms three days after the second batch of arrested L1s were fed (the first day of reproduction for worms grown at 15°C and 20°C) and progeny therefore differed in the maternal growth temperature and in the duration of maternal L1 arrest (1 vs. 2 days). Data were analyzed as described above for Maternal food availability and progeny reproduction.

### Maternal heat stress and progeny reproduction

Arrested L1s were transferred to NGM plates with excess food at 20°C and allowed to develop for 24 hours. Worms to be heat shocked were then transferred to at 35°C for 2 hours before being returned to 20°C, a treatment that is sufficient to significantly decrease lifetime fecundity but not to cause immediate mortality (data not shown). Control and heat shock worms were then allowed to develop for a further two days before progeny were isolated and analyzed as previously described. Progeny therefore differed in maternal heat shock exposure. Data were analyzed as described above for Maternal food availability and progeny reproduction.

### Maternal food availability and progeny dauer larvae development

Worms from a single cohort of arrested L1s were allowed to develop at a range of food concentrations until they were four days old at which point High, Medium and Low food maternal concentrations were selected as described above. Eggs were then isolated by hypochlorite treatment [32] and progeny allowed to arrest as L1s. These arrested L1s were transferred to 3.5 cm diameter plates (40–50 L1s/plate) containing 2 mL of dauer agar [33], 60 µl of dauer pheromone extract [19] and 20 µl of 2% OP50 *E. coli*. Plates were incubated at 25°C for two days and the proportion of dauer larvae on each plate determined. This assay was repeated three times. In all cases High maternal food availability was 10% w/v *E. coli*. Medium maternal food availability was 2.5% w/v *E. coli* for assays 1 and 3, and a combination of 2.5% and 5% w/v *E. coli* treatments for assay 2. Low maternal food availability was 1.25% w/v *E. coli* for assays 2 and 3, and a combination of 1.25% and 0.625% w/v *E. coli* treatments for assay 2. The proportions of progeny that developed as dauer larvae were analyzed by Kruskal-Wallis test.

### Progeny reproduction in stressful conditions

Dauer and non-dauer larvae were recovered from a dauer larvae assay (set up as described above). The lifetime fecundity and reproductive timing of these worms, was then determined, as described above, except that worms were maintained on dauer agar plates with 50 µl of 1% OP50 *E. coli* and maintained at 25°C. While this represents a smaller amount of food than that found on an NGM plate, it is still an excess of food for a single worm maintained on the plate for one day and other work has shown no differences between the lifetime fecundities or reproductive schedules of worms grown in these conditions in comparison to those grown on NGM plates (data not shown). Lifetime fecundity and reproductive timing of control worms, from the same cohorts of arrested L1s used in the dauer assay, was also determined on NGM plates with excess food at 25°C. Data were tested for normality and equality of variances and analyzed by ANOVA.

### Egg size

Worms, allowed to develop from L1 arrest at a range of food concentrations, High and Low food concentrations were then selected as described above and worms were then hypochlorite treated to isolate eggs [32]. Eggs were then transferred to dauer agar plates without food, photographed using a Moticam 2000 video camera (Motic, Wetzlar, Germany) and the cross sectional area determined in ImageJ [34]. This assay was repeated three times. In all cases High maternal food availability was 10% w/v *E. coli*. Low maternal food availability was a combination of 2.5% and 0.125% w/v *E. coli* for assay 1, 0.3125% w/v *E. coli* for assay 2 and 1.25% w/v *E. coli* treatments for assay 3. Egg size data were not normally distributed and were therefore analyzed by Mann Whitney U test.

## References

[1] Bernardo J (1996). Maternal effects in animal ecology.. Amer Zool.

[2] Räsänen K, Kruuk LEB (2007). Maternal effects and evolution at ecological time scales.. Funct Ecol.

[3] Benton TG, St Clair JJ, Plaistow SJ (2008). Maternal effects mediated by maternal age: from life histories to population dynamics.. J Anim Ecol.

[4] Roff DA (2002). Life History Evolution.

[5] Mousseau TA, Fox CW (1998). Maternal effects as adaptations.

[6] Lynch M, Ennis R (1983). Resource availability, maternal effects, and longevity.. Exp Gerontol.

[7] Gliwicz ZM, Guisande C (1992). Family planning in *Daphnia*: resistance to starvation in offspring born to mothers grown at different food levels.. Oecologia.

[8] Cleuvers MB, Goser B, Ratte H-T (1997). Life-history shift by intraspecific interaction in *Daphnia magna*: change in reproduction from quantity to quality.. Oecologia.

[9] Mitchell SE, Read AF (2005). Poor maternal environment enhances offspring disease resistance in an invertebrate.. Proc Biol Sci.

[10] Fitzsimmons JM, Innes DJ (2006). Inter-genotype variation in reproductive response to crowding among *Daphnia pulex*.. Hydrobiologia.

[11] Zhang TY, Bagot R, Parent C, Nesbitt C, Bredy TW, Caldji C, Fish E, Anisman H, Szyf M, Meaney MJ (2006). Maternal programming of defensive responses through sustained effects on gene expression.. Biological Psychology.

[12] Bashey F (2006). Cross-generational environmental effects and the evolution of offspring size in the Trinidadian guppy *Poecilia reticulata*.. Evolution.

[13] Grech K, Maung LA, Read AF (2007). The effect of parental rearing conditions on offspring life history in *Anopheles stephensi*.. Malar J.

[14] Mugabo M, Marquis O, Perret S, Le Galliard JF (2010). Immediate and delayed life history effects caused by food deprivation early in life in a short-lived lizard.. J Evol Biol.

[15] Barrière A, Félix MA, WormBook. ed. (2005a). Natural variation and population genetics of *Caenorhabditis elegans,*.

[16] Barrière A, Félix MA (2005b). High local genetic diversity and low outcrossing rate in *Caenorhabditis elegans* natural populations.. Curr Biol.

[17] Cutter AD (2006). Nucleotide polymorphism and linkage disequilibrium in wild populations of the partial selfer *Caenorhabditis elegans*.. Genetics.

[18] Cassada RC, Russell RL (1975). The dauer larva, a post-embryonic developmental variant of the nematode *Caenorhabditis elegans*.. Dev Biol.

[19] Golden JW, Riddle DL (1984). The *Caenorhabditis elegans* dauer larva: developmental effects of pheromone, food, and temperature.. Dev Biol.

[20] Hu PJ (2007). Dauer.. http://www.wormbook.org.

[21] Byerly L, Russell RL, Cassada RC (1976). The life cycle of the nematode *Caenorhabditis elegans*. I. Wild-type growth and reproduction.. Dev Biol.

[22] Hodgkin J, Barnes TM (1991). More is not better: brood size and population growth in a self-fertilizing nematode.. Proc Roy Soc Lond B.

[23] Harvey SC, Viney ME (2007). Thermal variation reveals natural variation between isolates of *Caenorhabditis elegans*.. J Exp Zool B: Mol Dev Evol.

[24] Harvey SC, Shorto A, Viney ME (2008). Quantitative genetic analysis of life-history traits of *Caenorhabditis elegans* in stressful environments.. BMC Evol Biol.

[25] Muschiol D, Schroeder F, Traunspurger W (2009). Life cycle and population growth rate of *Caenorhabditis elegans* studied by a new method.. BMC Ecol.

[26] Skorping A, Read AF, Keymer AE (1991). Life history covariation in intestinal nematodes of mammals.. Oikos.

[27] Bonte D, De La Pena E (2009). Evolution of body condition-dependent dispersal in metapopulations.. J Evol Biol.

[28] Wong A, Boutis P, Hekimi S (1995). Mutations in the *clk-1* gene of *Caenorhabditis elegans* affect developmental and behavioral timing.. Genetics.

[29] Bowerman B (1998). Maternal control of pattern formation in early *Caenorhabditis elegans* embryos.. Curr Top Dev Biol.

[30] Ward S, Carrel JS (1979). Fertilization and sperm competition in the nematode *Caenorhabditis elegans*.. Dev Biol.

[31] Van Voorhies WA, Fuchs J, Thomas S (2005). The longevity of *Caenorhabditis elegans* in soil.. Biol Lett.

[32] Stiernagle T, Hope IA (1999). Methods.. *C. elegans*: a practical approach.

[33] Viney ME, Gardner MP, Jackson JA (2003). Variation in *Caenorhabditis elegans* dauer larva formation.. Dev Growth Differ.

[34] Rasband WS (1997–2009). http://rsb.info.nih.gov/ij/.

